# Identification of ethics committees based on authors’ disclosures: cross-sectional study of articles published in the European Journal of Anaesthesiology and a survey of ethics committees

**DOI:** 10.1186/s12910-018-0289-y

**Published:** 2018-06-08

**Authors:** Davide Zoccatelli, Martin R. Tramèr, Nadia Elia

**Affiliations:** 1 Division of Anaesthesiology, Department of Anaesthesiology, Pharmacology and Intensive Care Medicine, Geneva University Hospitals, Rue Gabrielle Perret-Gentil 4, 1205 Geneva, Switzerland; 2 Faculty of Medicine, University of Geneva, Rue Michel Servet 1, 1211 Geneva, Switzerland; 3 Institute of Global Health, University of Geneva, Chemin des Mines 9, 1202 Geneva, Switzerland

**Keywords:** Ethics committee, Ethical approval, Scientific publication, Editorial policy, Fraud

## Abstract

**Background:**

Since 2010, the European Journal of Anaesthesiology has required the reporting of five items concerning ethical approval in articles describing human research: ethics committee’s name and address, chairperson’s name, study’s protocol number and approval date. We aimed to assess whether this requirement has helped to identify and to contact the referenced ethics committees.

**Methods:**

In this cross-sectional study, we analysed articles requiring ethical approval, according to the Swiss federal law for human research and published in the European Journal of Anaesthesiology in 2011. Ethics committees were searched through our institutional Internet access based on information provided in the articles. The last search was performed in November 2015. Numbers (%) of items reported, of ethics committees identified, and of those that confirmed having provided ethical approval are reported.

**Results:**

Of 76 articles requiring ethical approval, 74 (97%) declared it. Ethics committees’ names and addresses were mentioned in 63/74 (85%), protocol numbers in 51/74 (69%), approval dates in 48/74 (65%), and chairpersons’ names in 45/74 (61%). We could identify 44/74 (59%) committees; 36/74 (49%) answered our inquiry and 24/74 (32%) confirmed their role. Thirty-four of 74 articles (46%) reported all five items; in 25/34 (74%), we were able to identify an ethics committee, 18/34 (53%) answered our inquiry, and 15/34 (44%) confirmed their role. Forty of 74 articles (54%) reported ≤4 items; in 19/40 (48%), we were able to identify an ethics committee, 18/40 (45%) answered our inquiry, and 9/40 (23%) confirmed their role. Reporting five items significantly increased identification of ethics committees (*p* = 0.023) and their confirmation of ethical approval (*p* = 0.048). Twelve of 74 ethics committees (16%) were unable to confirm their role in approving the study.

**Conclusions:**

Even when details concerning ethical approval were reported in these studies of human research, we were unable to identify almost half of the ethics committees concerned. The reporting of five items, compared with reporting ≤4, was associated with facilitated identification of ethics committees, and increased the likelihood that they would be able to confirm the study’s approval. Future research should identify which information facilitates identification of, and contact with, ethics committees.

**Electronic supplementary material:**

The online version of this article (10.1186/s12910-018-0289-y) contains supplementary material, which is available to authorized users.

## Background

Protocols of clinical studies should be approved by an ethics committee before study launch to guarantee respect and protection of included persons, and to increase the quality of published research, as stipulated in the Ethical Principles of the *World Medical Association* (WMA) through the Declaration of Helsinki [1] since its second amendment (Tokyo 1975), and in the Ethical Guidelines of the *Council for International Organizations of Medical Sciences* (CIOMS) [2] since its first version (Geneva 1982). These texts have broadly influenced private and public institutions and are regularly updated and implemented in national and international regulations [3–8]. Thus published reports of human experimentation should explicitly state that a competent ethics committee has approved the study protocol. This has become mandatory for most scientific journals adhering to influential editors’ associations promoting integrity in research publication [9, 10], such as the *Committee On Publication Ethics* (COPE) [11] or the *International Committee of Medical Journal Editors* (ICMJE) [12].

Yet previous studies have highlighted that declarations of ethical approval are inconsistently reported in articles of human experimentation published in general medical journals [13], critical care journals [14], anaesthesia journals [15] but also in high impact factor journals [16–18]. In addition, research abstracts that are submitted to scientific meetings often lack declarations of ethical approval [19].

Moreover, recent cases of fraud in the anaesthesiology literature have demonstrated that simply reporting ethical approval is not sufficient. In fact, false declarations of ethical approval have often triggered institutional investigations. For example, Scott Reuben, who was the chief of the acute pain clinic at Baystate Medical Center, Springfield, Massachusetts, was first suspected of fraudulent practice when, during an institutional “research week”, he presented two studies that had not received formal approval by an ethics committee. His institution’s subsequent investigation brought to light Reuben’s fabrication of data and patients in more than 20 articles published over a decade [20–23]. Similarly in 2010, almost 90 articles authored or co-authored by Joachim Boldt, a German anaesthesiologist, had to be retracted from 18 peer-reviewed journals. Boldt’s massive fraud became obvious only when his former institution started an investigation into his research and discovered that most of his studies lacked formal ethical approval [24–28]. In these scandals, checking the approval of the research projects by a competent ethics committee was the first step of an investigation into research misconduct. Moreover, theses cases have highlighted that vague statements such as “this study received approval by the local ethical committee”, such as found in Boldt’s articles, were not adequate.

Based on these observations, the editorial board of the *European Journal of Anaesthesiology* (EJA), which had to retract eight of Boldt’s articles due to false declarations of ethical approval, decided in July 2010 to require authors to report five items in the first paragraph of the methods section: 1- name and 2- address of the ethics committee responsible for approval of the protocol, 3- protocol number, 4- name of the Chairperson of the ethics committee, and 5- date of the protocol approval. This change was made to discourage authors from submitting studies that lacked formal ethical approval and to allow for subsequent identification of the referenced ethics committee. The present study was designed to assess the efficacy of this measure.

Our primary objectives were to describe how many of the five items were reported in each article, the number (%) of ethics committees that could be identified and contacted by us through the information provided in the articles, and the number (%) of committees able to confirm their approval.

Our secondary objective was to examine whether reporting of all five items facilitated identification of the competent ethics committee and increased the likelihood that the committee confirmed approval of the study.

## Methods

### Study design

This cross-sectional study, comprising all original articles involving human research and published in the EJA in 2011, is reported according to the recommendations of the STROBE statement [29].

### Setting

These analyses were part of a doctoral thesis of DZ. The study was performed between 2015 and 2016 in the Division of Anaesthesiology, Geneva University Hospitals, Geneva, Switzerland, in close collaboration with the Editor in Chief of the EJA (MRT). All 12 issues of the EJA published in 2011 were hand searched by one author (DZ) who selected eligible articles regardless of content or study design.

### Eligible articles

According to the Swiss Federal Act on Research involving Human Beings [30], we classified articles into three categories. These choices were independent of the original authors’ appreciation. For category I articles, approval by an ethics committee was regarded as mandatory. These were experimental studies on human subjects and some non-experimental studies (for instance, observational studies based on non-anonymously collected data); these studies were further analysed. For category II articles, the need for approval by an ethics committee remained unclear or uncertain (for instance, experimental intubation studies performed on manikins). Category II articles were retained for the descriptive part of the analysis, but identification of, and contact with, ethics committees was not attempted. Category III articles consisted of animal studies, editorials, commentaries, narrative reviews, systematic reviews, meta-analyses, special articles, guidelines, book reviews, case reports and retractions. They did not require ethical approval and thus were not included.

### Variables

We extracted the following characteristics from all included articles: title, study design, first author’s name, country and affiliation as reported in the “Correspondence to author” section, and the country of the institution(s) where the study was performed.

For each article we extracted whether approval by an ethics committee was reported (yes/no). If this was the case, we extracted whether or not each of the five required items was reported. Specifically, we checked whether the authors reported the name of the ethics committee (yes/no), the address of the ethics committee (yes/no), the protocol number (yes/no), the name of the chairperson of the ethics committee (yes/no), and the date of ethical approval (yes/no). Requested items and examples of declarations of ethical approval are shown in Additional file 1.

We recorded whether or not an ethics committee could be identified (yes/no). Identification was considered successful when we were able to identify, based on the information provided in an article, a board that undoubtedly fulfilled the role of an ethics committee that was in relation with the study’s and/or the first author’s institution, and that provided contact details (e-mail and/or postal address). Searches were done in Google (https://www.google.ch) according to a standardised procedure (see Additional file 2). Search strategies included direct searches using designation and address of the ethics committee or the name of the chairperson, and indirect searches through the website of the institution where the study was conducted. Keywords used were “ethics”, “ethical”, “ethics committee”, “ethical committee”, and abbreviations (for instance, “REC” for Research Ethics Committee or “IRB” for Institutional Review Board). We also used appropriate non-English terms, for instance, *comité d’éthique* in French or *Ethisches Komitee* in German.

When we were able to successfully identify an ethics committee, we contacted them by email (or by letter, when only a postal address was provided) and asked whether or not they were the responsible board for that study, and whether or not they had approved the study. Reminders were sent to non-responders after two and again after three weeks. We classified the answers as “Yes, responsible and study approved”, “Yes, responsible and study not approved”, “Unclear whether responsible or study approved”, or “No answer”.

### Bias

In order to reduce the risk of bias, data extraction of the characteristics of the articles was performed by one author (DZ) and checked by another author (NE). Any discrepancy was discussed with the third author (MRT). A first attempt to identify ethics committees was performed by one investigator (DZ) between July and August 2015. In November 2015, a second investigator (NE) performed an independent web-search of ethics committees for all articles for which the first search was not successful.

### Study size

This study aimed to be mainly descriptive. Although we had no pre-hoc evidence regarding the prevalence of the reporting of the five items or of the impact of the completeness of reporting of the items on subsequent success of identification of ethics committees, we hypothesized that the complete 5-items reporting would be considered “effective” if it improved our ability to identify ethics committees by 30% (from 60 to 90%). For a bilateral test, alpha level of 5% with 80% power, a total of 64 articles were needed. Since the reporting of the 5-items requirement had been initiated in late summer 2010, and in order to find enough articles that did not yet fulfil the requirements (since recommendations take some time to be implemented), we decided to focus on the calendar year that immediately followed the editorial decision.

### Statistical method

Continuous characteristics of the journals are reported and summarized as means and standard deviations (SD) or medians and inter quartile ranges (IQR) depending on their underlying distribution, while dichotomous characteristics are described as numbers and proportions. We classified the articles into two groups according to the number of items reported (5 versus ≤4), and compared the proportion of ethics committees identified and the number of ethics committees confirming their role between the two groups, using a Chi2 test (alpha 0.05, bilateral).

## Results

### Eligible articles

**Fig. 1:**
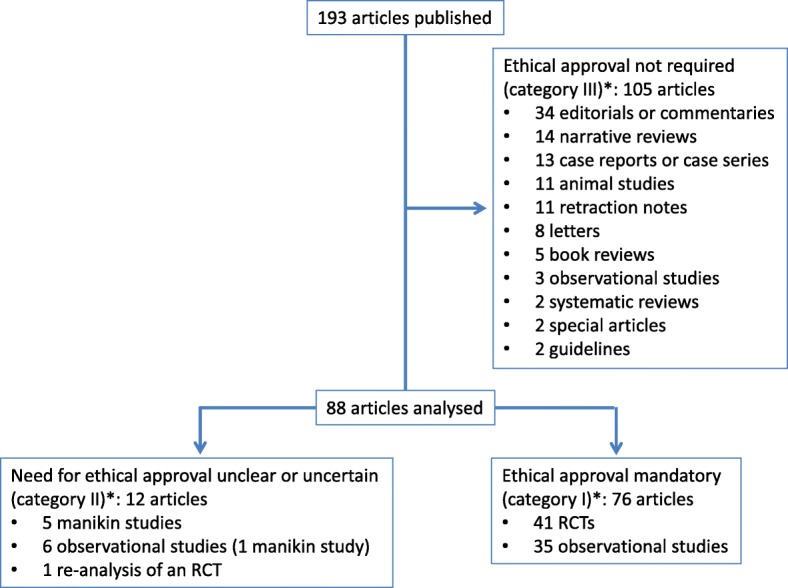
Flowchart of retrieved, excluded, and eventually analysed articles. Legend: RCT = randomized controlled trial. *According to Swiss Federal Act on Research involving Human Beings

In 2011, 193 articles were published in the EJA (Fig. 1). Of those, 105 were classified category III. Twelve were classified category II; most were volunteer studies performed on manikins. The remaining 76 articles were classified as category I; 41 were randomized controlled trials (RCTs) and 35 observational studies. These articles originated from 28 countries.

**Fig. 2:**
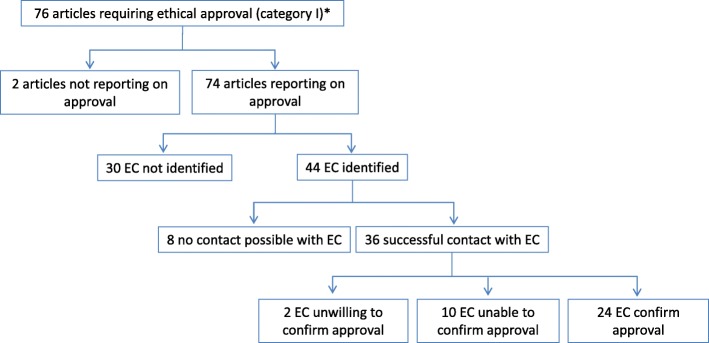
Identification of, and contact with, ethics committees and confirmation of approval of study protocols. Legend: EC = ethics committee. *According to Swiss Federal Act on Research involving Human Beings

Of the 76 category I articles, 74 (97%) reported on ethical approval, and two (3%) did not (Fig. 2). Of those that did not report on approval, one was a study of non-invasive measurements on healthy volunteers who were “personally well known” by the authors. The other was an observational study using a standardized general anaesthesia procedure in patients.

### Primary objectives

#### Reporting of the five items

Of the 74 articles that reported on ethical approval, 34 (46%) reported all five items and 40 (54%) reported four or fewer: 12 (16%) reported four, 11 (15%) reported three, eight (10%) reported two, three (4%) reported one item, and six (8%) reported none.

Name and address of the ethics committee were mentioned in 63/74 (85%) articles, protocol number in 51/74 (69%), date of approval in 48/74 (65%), and name of the chairperson in 45/74 (61%) articles.

#### Identification of ethics committees

We were able to identify ethics committees of 44/74 (59%) articles; 21 were RCTs, and 23 were observational studies. They originated from 21 countries. For 21/44 (48%) articles, we could easily identify the ethics committees using the information provided in the articles. For 20/44 (45%) articles, we had to search the web page of the authors’ institutions to identify the ethics committees. For the remaining three (7%) articles, we found the ethics committee “by chance” through a web-based PDF [31] with a list of postal addresses of 225 ethics committees from 33 countries; two of the “missing” ethics committees were listed in this document. The address of the third committee was similar to the one we were looking for and through some further research we were eventually able to identify it.

For 30/74 (41%) articles, we were unable to identify the responsible ethics committee (Fig. 2).

#### Contact with ethics committees and confirmation of their role

Of the identified ethics committees, 36/44 (82%; 49% of total) answered our inquiry (Fig. 2). Of the 8/44 (18%) that did not, we received a mail delivery system failure message for two (one for unspecified reasons, the other due to a full mail box).

Of the ethics committees that could be identified and that answered our inquiry, 24/36 (67%; 32% of total) confirmed having approved the study.

For 12/36 (33%) articles (11 ethics committees, one responding for two articles), the ethics committees were unwilling or unable to confirm their responsibility in approving the studies for a variety of reasons (see Additional file 3). Four committees (from Canada, Denmark, Spain and Sweden) reported having problems accessing or consulting their archives. Two (from the UK and Germany) questioned whether they were actually the ethics committee that had given approval of the study, or that ethical approval may not have been necessary (for instance, for audits). Two (both from Austria) answered that for reasons of data protection it was not possible for them to provide information on the studies. Two (both from France) informed us that our request had been transferred to their chairpersons; one also requested our CVs. Neither of the two committees subsequently provided us with any helpful information. One committee (from India) acknowledged having received our three letters but all at the same time and promised to answer our request but never did so. Finally, in one case (from Poland), we contacted an ethics committee, but unexpectedly received an answer from the first author of the article. He provided us with a scan of an apparent ethical approval of a different, larger study, from which, he explained, he had extracted the published data. According to him, the ethics committee had been informed of the procedure and did not require a separate approval.

### Secondary objectives

Of the 34 articles reporting all five items, we were able to identify 25 (74%) ethics committees. We received an answer to our inquiry from 18/34 (53%), and 15/34 (44%) confirmed that they were indeed the responsible committee for the study.

Of the 40 articles reporting four items or fewer, we were able to identify 19 (48%) ethics committees. We received an answer to our inquiry from 18/40 (45%), and 9/40 (23%) confirmed that they were indeed the responsible committee for the study.

Reporting five items significantly increased the likelihood that an ethics committee could be identified by us (74% versus 48%, *p* = 0.023), and increased the proportion of ethics committees that confirmed their role as the responsible entity (44% versus 23%, *p* = 0.048), but did not change the response rate of the ethics committees (53% versus 45%, *p* = 0.496) (Table 1).

**Table 1:** Identification of, and contact with, competent ethics committees, and confirmation of approval in relation to the number of reported items

		#items reported in article		
	Total	5	≤4	*p*-value
Articles requiring ethics approval (category I)^a^	74 (100%)	34 (100%)	40 (100%)	
Articles for which the competent ethics committee could be identified	44/74 (59%)	25/34 (74%)	19/40 (48%)	0.023
Articles for which the competent ethics committee could be contacted	36/74 (49%)	18/34 (53%)	18/40 (45%)	0.496
Articles for which the competent ethics committee confirmed approval of the study protocol	24/74 (32%)	15/34 (44%)	9/40 (23%)	0.048

^a^According to Swiss Federal Act on Research involving Human Beings

## Discussion

### Main findings

This study highlights four main issues. Firstly, only 44% of articles requiring ethical approval provided all five ethics items requested by the journal. This is not an unexpected result; the requirements were implemented only a few months earlier. Names and addresses of the ethics committees were most frequently reported, followed by protocol numbers. Names of Chairpersons responsible for approval were only marginally reported. Second, we were able to identify only 59% of ethics committees that were responsible for providing approval. Reporting of all five items in an article significantly increased the likelihood of identification of an ethics committee. Third, although 82% of the identified ethics committees answered our inquiries, only two thirds confirmed their role in the approval process. Again, reporting all five items increased the chance that the ethics committees confirmed their responsibility. Finally, reasons for not being able to confirm ethical responsibility ranged from logistic problems to legal constraints.

### What is already known on this subject

The frequency of reporting of ethical approval in published articles has increased over time [16, 18]. Also, a positive association was shown between the quality of the trials and the reporting of ethical requirements [32]. Previous studies have mainly focused on verifying whether or not a declaration of ethical approval was reported in published articles [13–18]. None of them actually verified whether the declarations were correct, whether the ethics committees existed, and whether they had given formal approval of the studies. Although Yank and Rennie had suggested, in 2002, that journals ought to implement in-house practices to try to improve the reporting of ethical approval [16], our study is the first, to our knowledge, that attempts to test whether or not the reporting of some specific items in the methods section of an article, facilitated subsequent identification of a competent ethics committee and increased the likelihood that an ethics committee had indeed approved a study. Our study shows that these expectations are only partially satisfied.

### What does this study add

This study brings to light two new relevant issues and raises questions. First, when authors publishing in the EJA in 2011 were willing to provide five specific items about ethical approval, the likelihood that the responsible ethics committee could be identified by us was increased; and once contacted, the ethics committee was more likely to confirm having given approval. It may be inferred that authors who are willing to provide several specific details on ethical approval are more likely to submit a study that has actually received approval, although our study does not prove this. Second, even when as many as five items are reported, it still appears difficult, or even impossible, to identify an ethics committee. This suggests that the items chosen by the editorial board of the EJA may not necessarily be the most relevant to guarantee successful identification of an ethics committee, or that further items should be added. For example, the chairperson of an ethics committee may change; a link to the committee’s website or an email address would probably be useful. So far, the most relevant items remain unknown. Finally, our study raises the question as to who should have the authority and should be able to contact an ethics committee? Should it be only the institution hosting the author, responsible for leading an investigation regarding potential misconduct? Or should this information also be accessible to journal editors who are responsible for the validity of published research? Or should there be free access to ethics committees and their approvals? This remains a matter of discussion.

### Strength and weaknesses of our analysis

This is a cross-sectional study of articles published during a one-year period in a peer reviewed, subspecialty journal. The choice of the year was made since it followed the implementation of the new editorial policy of the EJA that required details of ethical approval to be provided. The number of analysed articles is relatively small and the generalizability of our results remains unclear.

Two investigators classified the articles according to the need for ethical approval. Although the choices were not always straightforward, they did not depend on whether or not the authors of the articles reported on ethical approval. Also, since the assessment of the need for ethical approval may differ among countries, some of our choices, which were based on Swiss legislation [30], may be questioned. For instance, we classified some articles category III (articles not requiring approval), although their authors had declared having received ethical approval.

We may have missed some ethics committees due to our search method. However, two investigators conducted all searches independently, in various languages and using a variety of strategies and key words.

### Research agenda: where are we going from here

Comparison of our data with those published in previous studies confirms that the frequency of declaration of ethical approval has increased substantially in the last two decades [16–18]. Nonetheless, despite detailed information provided in most of these articles, half of the ethics committees could not be identified. Although reporting of five specific items ensures better identification of ethics committees, future research should aim to determine how this could still be improved. Joint research and collaboration among ethics committees, institutions and journal editors may be needed in order to identify which information is likely to be the most relevant. Regarding who should have access to this information remains a matter of discussion. Also, our study highlights that some ethics committees could improve their logistics capacity to track submitted research protocols.

## Conclusions

An article’s reporting of specific items related to ethical approval facilitates the identification of the ethics committee conferring approval and increases the likelihood that this approval can be confirmed. Nonetheless, identification of, and successful communication with, ethics committees remains a difficult task. Additional work is needed to better define the information that will facilitate successful identification of, and contact, with ethics committees.

## Additional files

Additional file 1: Requested items and examples of declarations of ethical approval. (DOCX 64 kb)

Additional file 2: Standardised procedure for identification of ethics committees. Google was used as a search engine (PPTX 74 kb)

Additional file 3: Selected problematic replies of ethics committees. (DOCX 116 kb)

## Abbreviations

EJA: European Journal of Anaesthesiology
RCT: Randomized controlled trial
