# Supplementary material for: Identification of ethics committees based on authors’ disclosures: cross-sectional study of articles published in the European Journal of Anaesthesiology and a survey of ethics committees
Source: BMC Med Ethics. 2018 Jun 8;19:57. doi: 10.1186/s12910-018-0289-y (PMC5994111; doi:10.1186/s12910-018-0289-y)
Supplement: Supplementary file 1 — Requested items and examples of declarations of ethical approval. (DOCX 64 kb) [file 12910_2018_289_MOESM1_ESM.docx]

| **Additional file 1:** Requested items and examples of declarations of ethical approval | |  |  |
| --- | --- | --- | --- |
|  |  |  |  |
| **# Items** | **Example** |  |  |
| 5 | Ethical approval for this study (**protocol number XYZ**) was provided by (**name of chairperson**), affiliation (**name of ethics committee**, **address of ethics committees**), on (**date of approval)** |  |  |
| 4 | Ethical approval for this study (**protocol number XYZ**) was provided by (**name of ethics committes, address of hospital**), on (**date of approval**) |  |  |
| 3 | Ethical approval for this study was provided by (**name of ethics committee**), affiliation (**address of hospital**), on (**date of approval**) |  |  |
| 2 | Ethical approval for this study was provided by (**name of ethics committee)**, affiliation (**name of hospital)** |  |  |
| 1 | After approval by the institutional review board (**protocol number XYZ**). |  |  |
| 0 | This study was approved by the institutional research board... With approval by the local ethics committee… Following ethical approval… |  |  |
|  |  |  |  |
