# Supplementary material for: Identification of ethics committees based on authors’ disclosures: cross-sectional study of articles published in the European Journal of Anaesthesiology and a survey of ethics committees
Source: BMC Med Ethics. 2018 Jun 8;19:57. doi: 10.1186/s12910-018-0289-y (PMC5994111; doi:10.1186/s12910-018-0289-y)
Supplement: Supplementary file 2 — Standardised procedure for identification of ethics committees. Google was used as a search engine (PPTX 74 kb) [file 12910_2018_289_MOESM2_ESM.pptx]

## Slide 1
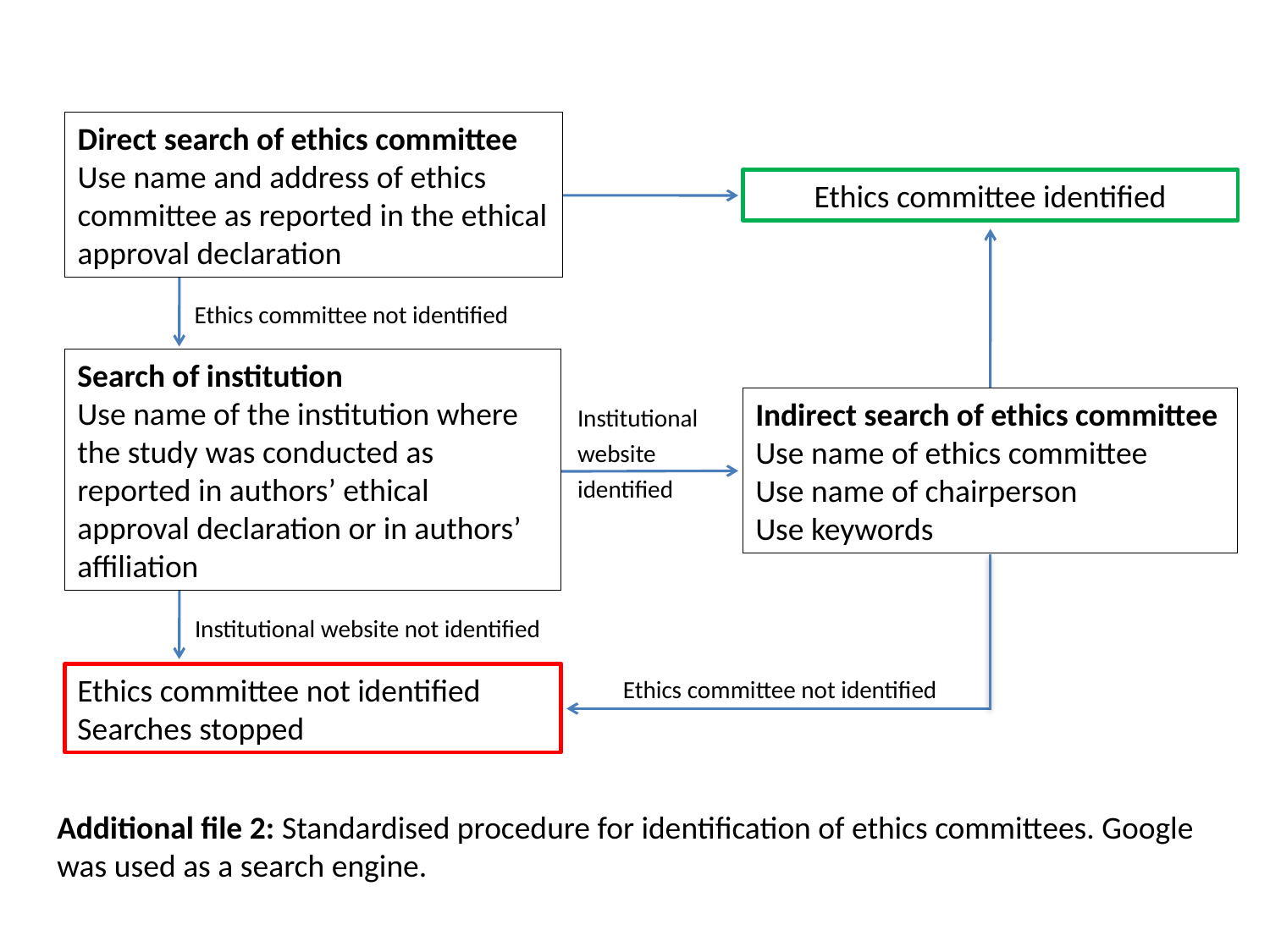

Direct search of ethics committee
Use name and address of ethics committee as reported in the ethical approval declaration
Ethics committee identified
Ethics committee not identified
Search of institution
Use name of the institution where the study was conducted as reported in authors’ ethical approval declaration or in authors’ affiliation
Indirect search of ethics committee
Use name of ethics committee
Use name of chairperson
Use keywords
Institutional
website
identified
Institutional website not identified
Ethics committee not identified
Ethics committee not identified
Searches stopped
Additional file 2: Standardised procedure for identification of ethics committees. Google was used as a search engine.
