# Supplementary material for: Identification of ethics committees based on authors’ disclosures: cross-sectional study of articles published in the European Journal of Anaesthesiology and a survey of ethics committees
Source: BMC Med Ethics. 2018 Jun 8;19:57. doi: 10.1186/s12910-018-0289-y (PMC5994111; doi:10.1186/s12910-018-0289-y)
Supplement: Supplementary file 3 — Selected problematic replies of ethics committees. (DOCX 116 kb) [file 12910_2018_289_MOESM3_ESM.docx]

| **Additional file 3:** Selected problematic replies of ethics committees |  |  |
| --- | --- | --- |
|  |  |  |
| **Original wording of ethics committees** |  | **English translation** |
|  |  |  |
| **A. Problems dealing with accessibility to, or consultation, of an ethics committee archive** | | |
| "Vi har begränsade sökmöjligheter i vårt diarium. Under år 2011 inkom två etikprövnings ansökningar med X.Y. som ansvarig forskare:  - Ansökan med diarienummer 2011/### och studietitel ”Inverkan av narkos på matstrupens funktion hos överviktiga och extremt överviktiga patienter. En studie på kraftigt överviktiga patienter före och under narkos” godkändes 2012-mm.dd. - Ansökan med diarienummer 2011/### och studietitel ” Inverkan av magsäcksreduktion kirurgi på övre magmunnen. En studie på kraftigt överviktiga patienter före och efter kirurgi.” godkändes med villkor 2012-mm-dd. - Under år 2010 inkom ingen etikprövningsansökan med X.Y. som ansvarig forskare." |  | "We have only limited search possibilities in our archive. Two study protocols submitted for ethics approval with X.Y. as the responsible researcher were archived in 2012: - Application with diary number 2011/### and study title "XYZ" was approved on 2012-mm-dd. - Application with diary number 2011/### and study title "XYZ" was approved with conditions on 2012-mm-dd. - In 2010, no ethical review was archived with with X.Y. as the responsible researcher." |
| "Le CER de l’établissement, alors sous la présidence de Dre X.Y., a été responsable de l’évaluation dudit projet. Ces données sont archivées et Dre X.Y. n’est malheureusement plus la présidente du CÉR depuis moult années. Il me serait difficile, voire impossible de répondre avec plus de détails aux étapes de l’évaluation de ce projet, lequel date de plusieurs années" |  | "The institutional committee, then under the chairmanship of the Dr X.Y., was responsible for the evaluation of the above-mentioned project. These data have been archived. Unfortunately Dr X.Y. is no longer the chairman of the reserach ethics committee. It would be difficult, if not impossible, to answer in more detail the stages of the evaluation of this project, which dates back several years." |
| "Regarding X.Y. it says in the article: "EC (name and address), decided that the study was in accordance with the ethical requirements and that formal approval by the committee was unnecessary because the study was purely descriptive." That might be the reason why I have not been able to find a case no. for that study." |  |  |
| "I am sorry to inform you that we are not able to find any EC approval titled (or similar) as the publication you sent us. Moreover Ms. *X.Y. (author)* does not work at this site any more so we are not able to ask her. We asked her colleagues and they are not able to tell us nothing about the publication's EC approval. I feel bad not been able to give you a positive answer so as a last verification I would like to ask you if you have any other document or information to send me to be able to check it again." |  |  |
|  |  |  |
| **B. Problems dealing with the identification of the competent ethics committee** | | |
| "Despite the Hospital X is mentioned as corresponding address in the paper we assume that the study was approved by the Ethics Committee in City Y (Ethik-Kommission des Fachbereichs Medizin der City Y University) which is the competent institution for the lead author." |  |  |
| "Many thanks for your letter dated 1st September requesting confirmation as to whether the following studies were approved by our ethics committees: 1) X.Y. (European Journal of Anaesthesiology 2011, Vol number: page numbers). 2) X.Y. et al (European Journal of Anaesthesiology 2011; Vol number: page numbers) Since December 2011, the Health Research Authority (HRA) is the appointing authority for all Research Ethics Committees (REC) in England and I am the appointing authority lead for the HRA. Unfortunately I was unable to locate the two studies on our database. We can search the database via a number of criteria including chief investigator name and study title but could not locate the exact studies you have referenced. It may be that the study was registered to another investigator of the study rather than Dr X.Y. If you are able to provide any other details such as REC reference number or any other reference number that would be helpful in locating this study. I note the paper refers to the studies as audits in which case REC approval may not have been sought and may explain why it states the approval was by the ‘research department of X.Y.Z. University Hospital’ rather than an ethics committee. Any further information you are able to provide would be helpful." |  |  |
|  |  |  |
| **C. Refering to specific regulations** | | |
| "Thank you for your inquiry. For data protection reasons it is not possible to provide information on studies. Please contact the author of the article" |  |  |
| "Ich darf Ihnen mitteilen, dass wir aus datenschutzrechtlichen Gründen keine Auskünfte an Dritte zu unseren Studien geben dürfen. Wir ersuchen Sie sich direkt mit den Prüfärzten bzw. den Antragstellern in Verbindung zu setzen." |  | "I must inform you that we shall not give information to third parties. Please contact the investigators of the study in secondary hospital in City X." (Ethics committee's own translation) |
|  |  |  |
| **D. Acknowledgement of receipt but no follow-up** | | |
| This is with reference to your letters dated September 1, September 15 and September 30 which I have received today regarding the article published by Dr *X.Y.* from our Institute and which was published in 2011 in the European Journal of Anaesthesiology, volume *number*, page *numbesr*.  We will look into your request and get back to you." |  |  |
| Votre demande à été transférée au Président de notre comité, je suis dans l'attente d'une réponse de sa part. Je vous la communiquerai dès que possible." |  | "Your request was transferred to the chair of our committee. I am waiting for his reply. I will come back to you as soon as possible." |
| "I have received your email regarding your research (see attached file that I've scanned); I have sent it to our Chairman Mr X.Y. Could you also send us the electronic version of your paper mail? And also the CV of the investigator? Thanks in advance. I am expecting our president answer to find out if we can analyze your query; I keep you informed" |  |  |
|  |  |  |
| **E. An author is replying instead of the ethics committee** | | |
| "I received information from my institutional ethics committee about your request concerning ethics committee approval for the study EJA 2011; *Vol number: pag numbers.* Please find attached scan of approval. This approval concerns bigger project dealing with muscle relaxation in morbidly obese patients undergoing general anaesthesia using rocuronium. The evaluation of intubation conditions after administration of rocuronium was a part of the study protocol. Since mentioned published data (*Vol number: page numbers*) were collected during performing bigger study following the accepted study protocol, the Local Ethics Committee received my explanation and I have not applied for separate approval." |  |  |
